# Photovoice and Being Intentional About Empowerment

**DOI:** 10.1177/15248399211062902

**Published:** 2022-03-14

**Authors:** Linda Liebenberg

**Affiliations:** 1 University of South Africa, Pretoria, South Africa

**Keywords:** Photovoice, voice in research, empowerment, collaboration, social change

## Abstract

The intent of Photovoice is to produce research in collaboration with communities, ensuring that research is relevant to community needs and critically facilitates change required to address these needs. Accordingly, Photovoice extends research for knowledge production, emphasizing research for social change. Consequently, Photovoice stands to make an important contribution to relevant and impactful knowledge production with health promotion research. However, if the intent of Photovoice as reflected in its theoretical underpinnings is not accounted for from the outset, the value of the approach may not be fully realized. This article considers what the theoretical underpinnings of Photovoice are, how this relates to issues of power and empowerment theory, and how voice can be better ensured within a process that is intentional about empowerment and representation.

This article considers what Photovoice is and how voice can be better ensured within the process, where voice is understood to mean the articulation of lived experience together with experiences of oppression, silencing, agency, and control. In addition, voice is recognized as a means of expressing resistance to dominant representations and a means of asserting power through more accurate representations of contextual risks and needs, as well as personal and contextual resources and strengths. Put simply, this article considers researcher orientation toward empowerment, voice, and shared power within Photovoice projects. It begins with a brief reflection on the challenges of power within public health knowledge creation. It then responds to these challenges by reviewing the theoretical underpinnings of Photovoice and the role of “voice” and power in Photovoice. Collectively, the article encourages consideration of whose voice is integrated into research and how. In addition, how do issues of power impact the integration of voice in research, thereby privileging some perspectives and silencing others? And finally, how does “voice” and its positioning in research projects vis-à-vis the recognition of power and positionality facilitate the social and systemic change needed by communities.

## Core Theoretical Traditions and Epistemologies in Public Health Research

For decades, people have called for the integration of diverse perspectives and experiences into public health research, improving representation of various groups (Baum, 1995). Such a move shifts knowledge production away from traditional epistemologies, such as positivism, empiricism, and reductionism, improving the balance of power within the research process (Green & Johns, 2019). Neoliberal knowledge economies continue to support traditional approaches (Rossiter & Robertson, 2014). Within these frameworks, researchers are seen as “experts,” positioned as knowledge holders and knowledge producers. In addition, research responds to research questions posed by credentialed “outsiders,” who also drive research design using quantitative methods, ensuring the objectivity of data and validity of findings (Rossiter & Robertson, 2014). Finally, research results are shared with limited audiences, predominantly practitioners, policymakers, and researchers (Marshall & Guenette, 2011). Ultimately, these approaches privilege the voices of “experts” at the expense of communities themselves, retaining practice and decision-making power outside of communities.

Moreover, where community involvement in research is increasing, this involvement is often critiqued for being tokenistic (Gibson et al., 2012). Rather than developing “user-controlled research” (Green, 2016, p. 3), community members are often positioned as consultants, whose input may or may not be used. This approach negates the value of authentic user engagement, once again impacting power dynamics in knowledge production.

## Orientations to Empowerment, Power, and Knowledge Production

Empowerment theory recognizes the broader social, political, and economic context in which people live and how this context works to oppress specific groups within society. Relatedly, empowerment theory considers experiences from a person-in-environment framework, recognizing the interdependence of people. Consequently, effective responses to the challenges people face need to co-occur at the intrapersonal, interpersonal, and community levels (East, 2016). Wallerstein and Bernstein (1988, p. 380) define empowerment as a process that “promotes participation of people, organizations, and communities in gaining control over their lives in their community and larger society. . . . empowerment is . . . [the] power to act with others to effect change.” Empowerment can therefore be seen as the facilitation of a person or community’s capacity to make intentional choices, resulting in actions that shape individual and community lives for the better (Perkins & Zimmerman, 1995), ordinarily through social policy and social change (Rappaport, 1987).

Research that fails to account for the positioning of power in knowledge production undermines the possibility of empowerment within the context of service delivery and lived experience. Weidenstedt (2016) discusses the paternalistic nature of empowerment, where one group sees itself as working to empower another group. Within qualitative and especially participatory action research (PAR), such paternalism may be hidden. The implication is that researchers may approach a context, seeing research participants (in qualitative research) or collaborators (in PAR) as powerless and believing that they as researchers have power to give. The implication is that “the less powerful individual is not only less powerful but also less competent and thus unable to direct his or her own destiny” (Weidenstedt, 2016, p. 7). This dynamic can result in feelings of subordination and disempowerment among research collaborators, undermining any possibility of genuine empowerment. Intentionally attending to PAR principles within the application of Photovoice, I argue that researchers can respond to these concerns in public health research.

These various considerations need to extend throughout the research process. Questions such as “who holds power to decide what topic should be researched and how? Who has the expertise to determine the best way in which to research this topic? And finally, who has insights on various audiences with whom to share findings?” should guide researcher–collaborator interactions.

## Empowerment and Photovoice: Revisiting the Role of Critical Pedagogy, Feminist Theory, and Par

In their first publication, Wang and Burris (1994) remind us that

The goal of photo novella [i.e., Photovoice] is to use people’s photographic documentation of their everyday lives as an educational tool to record and to reflect their needs, promote dialogue, encourage action, and inform policy . . . Photo novella is designed to include new voices in policy discussions by facilitating collective learning, expression, and action. (pp. 171–172)

The aim of including “new voices” in policy discussions sits literally and figuratively at the heart of this approach. However, this goal’s attainment is underpinned by the research team’s intentional implementation of Photovoice methods via group facilitation. This is possibly why Wang and Burris so carefully wove the three underpinning theories of critical pedagogy (learning), feminism (expression), and PAR (action) into their approach. In addition, they bookended the value of Photovoice as “changes in consciousness and to informing policy” (p. 172), highlighting the importance of critical pedagogy as part of participation and social change as part of action in this research approach. It is not the mere handing of cameras to collaborators and hosting an exhibition that changes broader social thinking and doing. Instead, the interwoven and cyclical implementation of these three foundational components feeds into a knowledge-for-change cycle (Liebenberg, 2018).

Intentional use of critical pedagogy challenges superficial insights into personal experiences, facilitating a richer understanding of how experiences are situated within and shaped by larger structural systems. The implied value of asking people to discuss their life experiences has been questioned by various authors (cf. Teachman & Gladstone, 2020), especially when considering the positioning of voice within larger socio-economic and historical systems (Kuntz, 2015). Many structural aspects of marginalization limit the extent to which people reflect critically on their experiences and related contextualizing factors. Consequently, qualitative data can be limited to cursory understandings of experiences and may result in the perpetuation rather than alleviation of contextual constraints (St. Pierre, 1997). Integrating the principles of critical theory into dialogue about lived experience can address these limitations, supporting the elicitation of richer data and informing our knowledge-base and related policy and practice implementation more impactfully. To facilitate richer insights, Wang and Burris used the act of making photographs and discussing them in a group as advocated for by Paulo Freire.

Freire (1973/2002) is widely regarded as the architect of critical pedagogy, basing his approach on critical theory. Acknowledging the many and ever-evolving critical theories, authors such as Howell (2013) argue that significant similarities exist across these various theories. Specifically, critical theories seek to challenge ruling ideologies to promote equality and liberty. Critical theories seek to uncover the

positions of power in between institutions, groups and individuals as well as . . . the rules regulations and norms that prevent people from taking control of their lives; the means by which they are eliminated from decision making and consequently controlled. (Howell, 2013, p. 77)

Accordingly, understanding people requires understanding their current and historical context together with the narratives used in these contexts to maintain the oppression of various groups. By identifying these interrelated components, communities can understand the mechanisms of their oppression and ways of asserting their agency, moving toward equality and liberty.

Building on this, Freire argued that through group conversation, people’s expertise emerges, and knowledge is co-constructed. Such “dialogue,” however, requires equality and mutual respect among participants to be effective. In addition, to gain a meaningful understanding of their social reality, people need to engage in an intentional process of reflection with their environment, a process Freire called “praxis.” Through “praxis,” Freire believed people achieve “conscientization,” critical awareness of their positioning within the larger systems shaping their social reality. This awareness can then be used to inform subsequent action and the changing of social reality. Here, Freire believed photographs could function as a mirror to culture and society and facilitate conscientization when discussed collectively. Collectively, through the group discussion of photographs, a richer understanding of lived experience is developed.

Subsequently, numerous researchers have expounded Freire’s argument that photographs facilitate greater insight into lived experience. In reviewing the literature, Rose (2016) surmises that integrating photographs into discussions provides research participants and collaborators with an opportunity to reflect more deeply on the taken-for-granted, everyday aspects of their lives. As argued elsewhere (Liebenberg, 2009), the act of pausing to document a moment or a location in a photograph generates questions about the importance of that aspect of life: Why did it need to be documented? Rose (2016) concludes that the process of making photographs of daily life creates a distance for participants and collaborators from everyday events, prompting reflection on experiences. This reflection, in turn, is brought back into the group discussion, where collaborators generate new insights into their collective experiences via group discussions. To summarize, it is the creative use of a facilitation tool (such as photography) to reflect more deeply on everyday experiences, combined with the intentional use of group work to develop a critical understanding of these experiences as situated within broader socio-economic structures, that are at the center of the Photovoice process.

This process of critical reflection and education aligns with certain goals of feminist theory. The feminist theory initially used in Photovoice was intended to integrate collaborators as full partners in the research and advocacy process, and foreground the subjective experiences and core concerns in collaborators’ lives in policymaking (Wang et al., 1996). Through the pedagogical process of critical reflection and dialogue, collaborators can delve beyond superficial experiences and needs, and identify the larger issues that sit beneath their experiences that need to be addressed or require change.

While Wang and Burris never specified what feminist theory they were using, Latz (2017) provides a compelling argument for standpoint theory as the theoretical basis for the feminist component of Photovoice. With its roots in a Marxist critical paradigm, standpoint theory mirrors critical theory. It too focuses on the attainment of social justice via the exploration and understanding of systemic oppression. Standpoint theory

argues that group location in hierarchical power relations produces shared challenges for individuals in those groups. These common challenges can foster similar angles of vision leading to a group knowledge or standpoint that in turn can influence the group’s political action. (Collins, 1990, p. 201)

As with critical theory, understanding how personal perspectives (“positionality”) are shaped by broader social structures is central to challenging power (Smith, 1987).

Reflecting on this theory, Latz (2017) argues that integrating collaborators as full partners throughout the Photovoice process, with expertise on their own lives, “places participants’ standpoints at the centre of the work” (p. 35). Moreover, the images created in the process of Photovoice reflect the positionality of research partners. Furthermore, the group dialogue of these images underpins the active exploration of experience and participant knowledge, illuminating participant praxis and the intersectionality of their experiences. Consequently, “inequities, stereotypes, stigmas, and marginalization can be amplified and reconstituted through Photovoice work” (p. 37).

Surrounding these two interacting theoretical components is PAR. As with critical theory and feminist theory, there are many versions of PAR, called by various names. Again, however, there are core components of underpinning PAR theory: the goals of system improvement and emancipation achieved through research, action, and education. In addition, cutting across various PAR approaches is the centrality of community, where information and capacity are negotiated and shared. Minkler and Wallerstein (2008) explain that PAR provides an approach to research rather than a method; methods are situated within this theoretical approach and implemented in ways that respect communities’ capacity, integrating community members as full research collaborators.

Consequently, the skills that everyone (academics and community collaborators alike) brings to the research are fully integrated into the process from the beginning of the project. PAR studies, including those using Photovoice, are not conceptualized and designed in the offices of academics. Data are not owned by academics, implying that they are also not analyzed in isolation from the communities who own them. And the dissemination process is not determined by academics. Full collaboration is initiated at the very beginning of projects. This collaboration informs issues of concern to the community and serves to identify those best positioned to speak to the topic, providing insight into how it is experienced. Full collaboration also means that research partners are engaged in the analysis of data and dissemination of findings. These latter components, in particular, need to be negotiated within the entire team. It cannot be assumed that academic researchers will complete the analysis process without meaningful input from the rest of the group. Similarly, it cannot be assumed that merely presenting photographs in some form of an exhibition will effectively convey “voice” to the intended audience. Consequently, as with data production, analysis and dissemination activities should be intentionally and carefully considered (Latz, 2017; Liebenberg et al., 2020; Mitchell et al., 2017).

### Current Shortcomings in the Implementation of Photovoice

As previously stated, central to the intent of Photovoice is the integration of “new voices” in knowledge production, sharing, and related social change. Moreover, the successful implementation of Photovoice requires authentic collaboration between researchers and community partners throughout the research process. This collaboration needs to consistently address power issues to ensure that the voice of community research collaborators remains at the forefront of the process, supporting empowerment and continued community-driven social change. While many projects integrate community input into the design of projects and the data gathering component, we read less about how research collaborators conduct data analysis. Similarly, we read little about the intentional use of dissemination activities to bring about social change effectively. While calls to address these limitations in the use of Photovoice are not new, they remain largely unaddressed.

Data analysis is a critical component of intentional research partner empowerment. Yet, little practical guidance is offered on the act of participatory data analysis, especially where research collaborators are already giving much of their time and energy to the process (Reich et al., 2017). Consequently, in the publication of findings, researchers often limit their discussion of collaborative data analysis engagement to the actual production of data, arguing that the narrative accompanying photographs amount to participant data analysis. Given the earlier discussion of language in critical pedagogy, we can see how that discussion of photographs is not necessarily data analysis. In many ways, especially earlier narratives serve as core data. Additional reflection on these core data is required to identify core themes that cut across collaborators’ experiences. As discussed elsewhere (Liebenberg et al., 2020), Shaffer’s (1986) SHOWeD method provides a helpful framework that can be used to guide the focus of group discussions from data production through to analysis.

Ironically, Photovoice lends itself well to a more engaged form of analysis. The group nature of Photovoice establishes a strong basis from which collaborators can code data, generate themes, and even develop theories from their discussion and exploration of experiences (see, for example, Liebenberg et al., 2020). In the review of their narrative data, creative approaches can be used by collaborators to generate codes. Researchers/facilitators on the team can then use facilitation techniques such as the SHOWeD questions (Shaffer, 1986), guiding community collaborators to attach codes to the data, as discussed in the group sessions, together into themes and theories. In short, collaborators’ engagement in the actual analysis of data can be creatively supported by considering the enactment of data analysis and how this intersects with the research context (Coad & Evans, 2008).

As with data analysis, public sharing of findings to foster change is seldom discussed in detail. The predominant approach to this sharing is a public exhibition of photographs to which key stakeholders and often the public are invited. However, the reader is often left wondering whether stakeholders of relevance to the issue were invited, did they attend, and if so, what new knowledge and interventions did they leave with. Intentional consideration of how findings are shared and with whom has important implications for amplifying voice and empowering research collaborators.

As with other research approaches, Photovoice research can produce multiple results. It is valuable to work through these findings and identify who should hear what results and why as a team. Creating this dissemination road map can enable the team to better identify who to share results with and, importantly, how best to do this so that the dissemination of knowledge leads to relevant and impactful action. At times, there can be value in sharing results via a public exhibition (see, for example, De Lange et al., 2016). Other times, however, it may prove more impactful to share specific findings with a limited audience in a more targeted manner (see, for example, De Lange, 2017). Indeed, such targeted sharing may also expand the networks of community collaborators, supporting their social change work moving forward. Either way, for “the voice” of research collaborators to be amplified and relatedly heard, those audiences that can contribute to actual change and how findings are shared with them need to be strategically planned (Latz, 2017; Mitchell, 2015; Mitchell et al., 2017).

Without careful consideration of how knowledge is shared and how this sharing strategically integrates its own pedagogical components targeting audiences and knowledge users, it will not only preclude a project’s impact on policy and social change but may additionally risk disempowering collaborators. Implementing a research program with community collaborators on the promise of change that then fails to engage in knowledge-to-action cycles intentionally (Bober, 2011; Wang et al., 2004) raises ethical questions regarding researchers in managing and meeting community expectations regarding change (Mitchell, 2015; Wang & Redwood-Jones, 2001). One might even consider such situations exploitative in light of what research collaborators have contributed to the research.

## Implications for Researchers: Photovoice and Being Intentional About Empowerment

Applying Weidenstedt’s (2016) argument to Photovoice, within an academic context, researchers run the risk of assuming the role of the “empowerer,” deciding what is needed or how. Rather, these decisions are better made collaboratively with community partners. Within this process, researchers should not relinquish their participation. Rather, through bidirectional and authentic negotiation, academics and community partners can intentionally highlight the strengths and resources within the larger group. They can then work collaboratively to integrate these resources into the entire research process. In this way, community partners can be effectively situated both as experts in their own lives and as community advocates to promote positive social change. Engagement in fieldwork provides community partners with an opportunity to experience how research for social change requires their expertise regarding how and what data are gathered to be successful. In addition, engagement in the data analysis supports their confidence in the knowledge they are sharing. Finally, participation in the dissemination planning ensures that findings are shared with all relevant knowledge users and that community partners understand the full extent and intention of the knowledge sharing. Underpinning the impact of this process is the effort on the part of the researcher to account for their own power.

An empowerment process that accounts for power paradoxes can manifest in community collaborators being aware of and owning their knowledge and expertise. Concomitantly, through sustained and increased knowledge-sharing opportunities, they can become effective advocates for the social change their community requires. Finally, the combination of expertise with confident and skilled advocacy enhances the probability of achieving impactful change. Collectively, this is how the implementation of Photovoice accounts for the three underpinning theoretical components and effectively ensures “participant” or collaborator voice.

## Conclusion

Voice in Photovoice is not a given. However, the potential of voice and its power in Photovoice is significant. To ensure that research collaborators who engage in these research endeavors with academic research teams can and do meaningfully access the voice component of Photovoice, however, requires a considered and intentional approach to applying these methods in the field. Underpinning an effective approach requires that teams critically consider what it means to conduct a PAR study and how aspects of power and empowerment are situated and managed throughout the process. These considerations and how they impact the doing of the project scaffold authentic engagement in the knowledge production and knowledge mobilization components of the research. Equally important, a more considered approach would include critical reflection on the sharing of knowledge: who, how, and why would feature in dissemination planning, as would the question of impact. Collectively, this more engaged implementation of Photovoice can result in the authentic inclusion of “new voices in policy discussions” (Wang & Burris, 1994, p. 172).
